# Microencapsulation-based cell therapies

**DOI:** 10.1007/s00018-022-04369-0

**Published:** 2022-06-08

**Authors:** Safiya Naina Marikar, Assam El-Osta, Angus Johnston, Georgina Such, Keith Al-Hasani

**Affiliations:** 1 Epigenetics in Human Health and Disease, Department of Diabetes, Central Clinical School, Monash University, Melbourne, VIC 3004 Australia; 2 Monash Institute of Pharmaceutical Sciences, Monash University, Parkville, VIC 3052 Australia; 3 School of Chemistry, The University of Melbourne, Parkville, VIC 3010 Australia

**Keywords:** Cell reprogramming, Microencapsulation technologies, Tissue repair, Type 1 diabetes, B-cell transplantation, Cardiac, Liver, Neural tissue repair

## Abstract

Mapping a new therapeutic route can be fraught with challenges, but recent developments in the preparation and properties of small particles combined with significant improvements to tried and tested techniques offer refined cell targeting with tremendous translational potential. Regenerating new cells through the use of compounds that regulate epigenetic pathways represents an attractive approach that is gaining increased attention for the treatment of several diseases including Type 1 Diabetes and cardiomyopathy. However, cells that have been regenerated using epigenetic agents will still encounter immunological barriers as well as limitations associated with their longevity and potency during transplantation. Strategies aimed at protecting these epigenetically regenerated cells from the host immune response include microencapsulation. Microencapsulation can provide new solutions for the treatment of many diseases. In particular, it offers an advantageous method of administering therapeutic materials and molecules that cannot be substituted by pharmacological substances. Promising clinical findings have shown the potential beneficial use of microencapsulation for islet transplantation as well as for cardiac, hepatic, and neuronal repair. For the treatment of diseases such as type I diabetes that requires insulin release regulated by the patient's metabolic needs, microencapsulation may be the most effective therapeutic strategy. However, new materials need to be developed, so that transplanted encapsulated cells are able to survive for longer periods in the host. In this article, we discuss microencapsulation strategies and chart recent progress in nanomedicine that offers new potential for this area in the future.

## Background

Today, many diseases are not adequately treated by the conventional therapeutic methods based on the oral administration of drug substances. Microencapsulation offers an attractive cell therapy strategy with demonstrated feasibility and efficacy, especially in diseases where minute-to-minute regulation of a metabolite is necessary such as in the case of diabetes. The concept is simple: Cells that naturally secrete a bioactive substance are wrapped, or encapsulated, in a semi-permeable membrane. Following encapsulation, these cells are implanted in patients to allow in situ release of the desired substance. An essential aspect of this technology lies in the properties of the encapsulation membranes used. These should allow the free diffusion of small molecules, such as the nutrients and oxygen needed for the survival of encapsulated cells as well as the secretion of therapeutic proteins. On the other hand, molecules of high molecular weight, such as antibodies, as well as host immune cells must not be able to reach and destroy encapsulated cells.

## Key considerations for cell microencapsulation

Cells can be encapsulated for implantation by two primary means, entrapment within a gel matrix [1–3], or direct attachment of a thin-semi-permeable membrane onto the surface of the cell [4]. Here, we will focus on the engineering requirements for forming thin semi-permeable membranes on cells, as a number of excellent reviews have covered the formation of matrices for larger implants [5, 6].

**Fig. 1:**
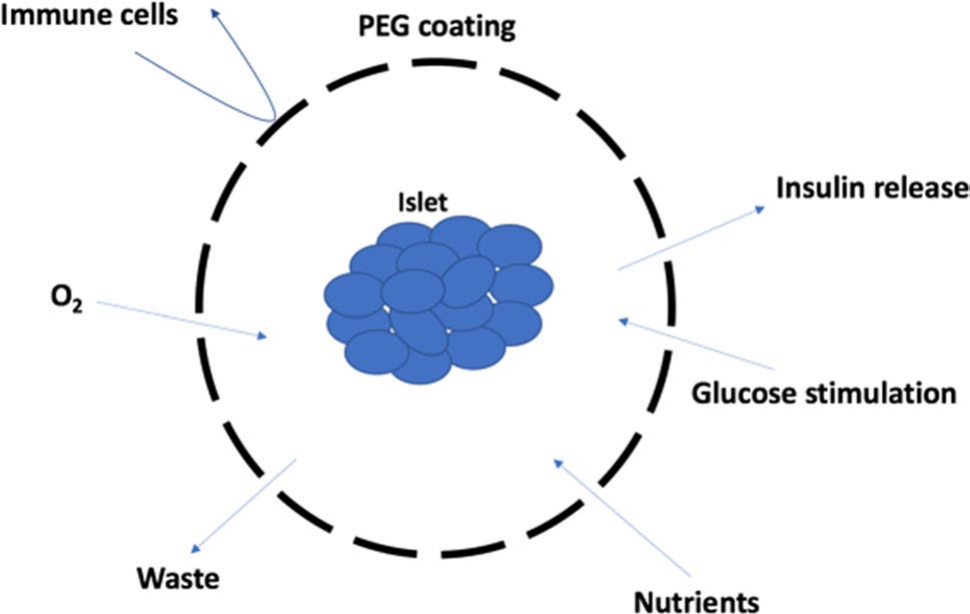
A prototypic pancreatic islet inside a semi-permeable and biocompatible membrane (PEG). This physical membrane blocks the passage to high-molecular-weight compounds (immune cells, antibodies) whilst ensuring the free release of glucose, insulin, oxygen, and nutrients necessary for the survival of the transplanted islets

The encapsulating polymer must provide a barrier to prevent the immune system from recognising the foreign cells. The polymer shell acts as a steric barrier which prevents the host T cells from recognising foreign antigens on the surface of the transplanted cells, but also prevents the host antibodies from binding to the transplanted cells. In addition to providing a steric barrier against the immune system, the polymer shell must be a semi-permeable membrane that allows the transport of key nutrients into the encapsulated cells, whilst also allowing waste products and the desired therapeutic molecules to diffuse out of the implant (Fig. 1).

The thickness of the shell around the cells directly affects the diffusion of molecules through the membrane, with thicker membranes slowing diffusion. As a general rule, thinner membranes are more desirable, as they allow rapid diffusion of nutrients to the cells as well as rapid removal of waste products. However, for the membranes to be effective, they must completely encapsulate the whole cell. Any small gap in the membrane will compromise the whole encapsulation process and make the transplanted cell vulnerable to rejection. For this reason, uniformity of coverage is crucial, and often, coating thickness needs to be increased to ensure complete encapsulation. Another consideration for encapsulating cells within a thin shell is ensuring the cells are fully differentiated and no longer dividing. Unlike large matrices that encapsulate multiple cells where there is room for cell division, the thin shells directly coated onto the cell cannot accommodate cell division. If the cells divide, large patches of the cell membrane will be uncovered, and the implanted cells will rapidly be recognised by the immune system.

Additional factors influence the efficacy of encapsulation. One of these factors involves providing the optimal matrix cues for cell encapsulation. In the case of encapsulated islet cells, the diffusion of glucose into the transplant is required to trigger insulin production [7]. Glucose is a small molecule that will readily diffuse through semi-permeable membranes along with the key nutrients required to keep the cells alive. The molecular weight of insulin is quite low (5.8 kDa), which means that it will diffuse through a relatively dense matrix. If higher molecular weight therapeutics are produced by the implant, then less dense matrices are required; however, this can increase the chance of diffusion of undesirable proteins into the transplanted cells. Another consideration for the diffusion of molecules through the membranes is charge. Insulin has an isoelectric point of 5.3, meaning that it has a negative charge at physiological pH. This means that it can electrostatically interact with positively charged membrane materials, which will prevent it from diffusing effectively through the membrane.

The requirements for membranes that encapsulate single cells or small cell clusters are quite different from the matrices required to support a larger number of cells in a macroscopic implant. When encapsulating single cells, it is normally desirable to make the membrane as thin as possible, with a range of diameters investigated [3, 8–11]. To control diffusion through thin membranes, the density of the membrane needs to be sufficient to prevent the diffusion of large proteins, such as immunoglobulins, fibrinogen, and complement, which range in size from 150 to 900 kDa [6]. In contrast, macroscopic matrixes typically provide a much thicker barrier between cells, typically tens of microns. The large distances that the molecules need to diffuse means the matrix is typically low density to facilitate the diffusion. These highly porous matrices are typically poor at controlling the diffusion of undesirable molecules, and so are often coated with a thinner outer layer, similar to that used for encapsulating single cells.

Cell encapsulation can take on different forms, single-cell encapsulation or encapsulation of small cell clusters as covered in these relevant reviews [6, 12–15]. Single cell encapsulation offers a defined way of engineering materials for implants. Diffusion of nutrients and waste from the cell is simple to achieve, as the diffusion distance out of the implant is small. Encapsulation of small clusters of islet cells can be achieved in a similar way to the encapsulation of single cells; however, additional thought needs to be given to the size of cluster encapsulated. If the cluster is too large, diffusion of nutrients in and waste out is retarded, leading to necrosis of the cells in the centre of the cluster.

## Mechanics of microencapsulation

### Polymer composition

#### Natural polymers

**Fig. 2:**
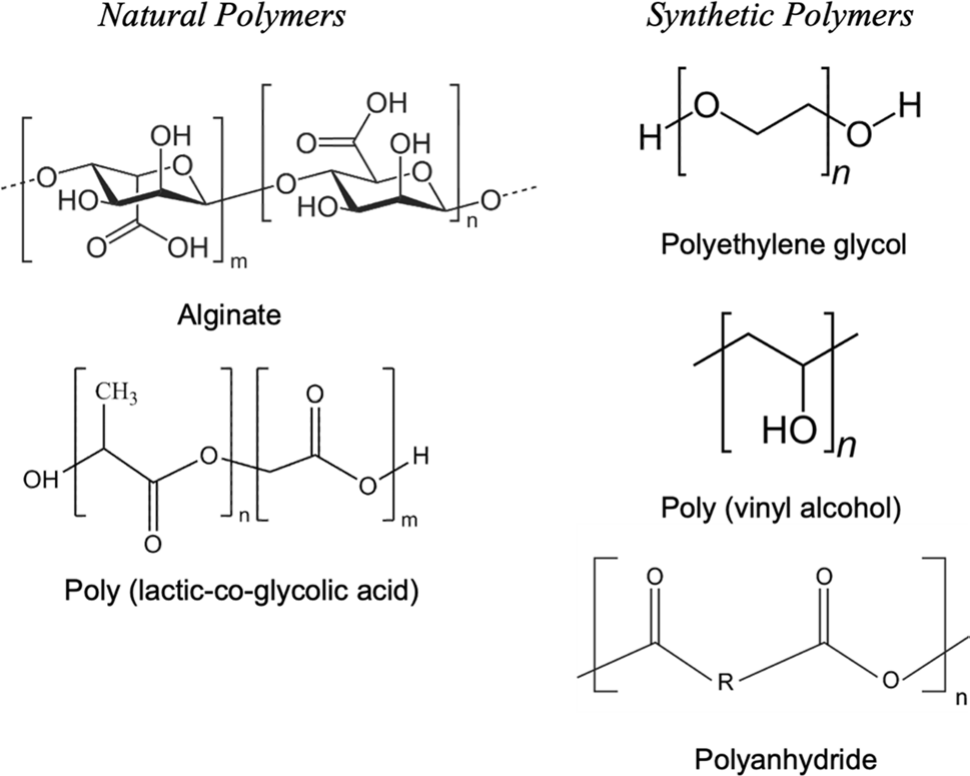
Chemical structure of natural and synthetic polymers used in cell encapsulation. With respect to natural polymers, their advantages include: bioactivity and biocompatibility; however, the key disadvantages include weak mechanical strength, immunogenicity, and uncontrolled rate of degradation. Synthetic polymers on the other hand, are easy to synthesis, have established structures, non-degradable, and possess tunable properties. Conversely, they lack cell adhesion sites

Naturally derived polymers have generated interest in cell encapsulation for many years mainly due to the inherent biocompatibility of these materials (Fig. 2). One of the most commonly used polymers for cell encapsulation is alginate (ALG), either in isolation or in combination with other polymers or specific biological molecules such as growth factors. ALG is biocompatible, shows low toxicity, is easy to gelate, and is cost-effective. Under mild pH and temperature, ALG can rapidly cross-link in the presence of divalent cations such as Ca^2+^. In recent work, researchers have investigated strategies to improve the ability of this polymer to mimic a natural ECM matrix. In one such paper, researchers combined human adipose tissue-derived ECM hydrogel with ALG matrix to form hybrid interpenetrating network microparticles for encapsulation of islet cells [16].

Another polymer that has generated significant interest for a range of biomedical applications including encapsulation is poly(lactic-co-glycolic acid) PLGA. This polymer is of interest due to its FDA approval and tuneable degradation under biological conditions with degradation products that are already produced in vivo. However, studies have also demonstrated that PLGA can cause inflammation and toxicity [17]. In a recent study, PLGA/Pluronic membranes were synthesised and converted into envelope-shaped pouches with one side open. The envelope was then incubated with Mesenchymal Stem Cells for 1 h at 37 °C, and then, the envelope was sealed to produce macro-encapsulated PLGA depots. The potential of these materials was investigated for the treatment of liver disease. The results showed increased survival of encapsulated MSC of over 28 days as compared to 1 week of direct tail vein injection.

Whilst being amongst the most commonly utilised polymers for microencapsulation, natural polymers such as alginate for the encapsulation of cells is not without their issues, namely the instability of the extraction process, leading to variations in the purity of the product [18], as well as the degradation that occurs when transplanted in vivo which leads to fibrosis. Pericapsular fibrosis results from the adhesion and aggregation of cells (macrophages, fibroblasts) on the surface of the microcapsule membrane. The fibrosis is problematic, because it eventually clogs the pores of the membrane and prevents the diffusion of oxygen, nutrients, and metabolites through the membrane, which compromises the function and viability of encapsulated cells [19].

#### Synthetic polymers

The use of more stable synthetic polymers can be used to overcome the inherent degradability of natural systems. Synthetic polymers offer the ability to precisely control their functionality, molecular weight, and morphology, as well as minimise their interactions with the immune system (Fig. 2). One attractive option to design such materials is poly(ethylene glycol) (PEG) as it has high biocompatibility and low toxicity, and is known to minimise non-specific interactions within a biological environment. In recent work, PEG diacrylate microcapsules were synthesised with tunable degradation based on the incorporation of a cleavable sequence GGLGPAGGK [20]. These microcapsules could be used to incorporate neural stem cells (NSCs) or the combination of NSCs and endothelial cells (ECs) [20]. These materials were investigated to improve intracerebral implantation of NSCs to treat stroke, a procedure which as of yet remains inefficient. To provide an additional layer of protection, these microcapsules were suspended in an extracellular matrix (ECM). This combination formulation showed enhanced delivery and proliferation of NSCs in the injection site.

Another synthetic polymer that has generated interest for encapsulation is poly(vinyl alcohol) (PVA) as it has high biocompatibility, high water incorporation, and low interactions with biological materials. In one recent study, PVA was used to encapsulate bone marrow mesenchymal stem cells (hMSCs) by the cross-linking of vinyl ether acrylate-functionalised PVA with thiolated vinyl ether arylate-functionalised PVA through a Michael-type cross-linking reaction [13]. They also demonstrated the co-encapsulation of growth factors to tune the behaviour of the encapsulated cells. This synthesis was conducted using microfluidics allowing control over nanoparticle properties by tuning the flow speeds of the different components [21].

#### Attachment to cell surface

**Fig. 3:**
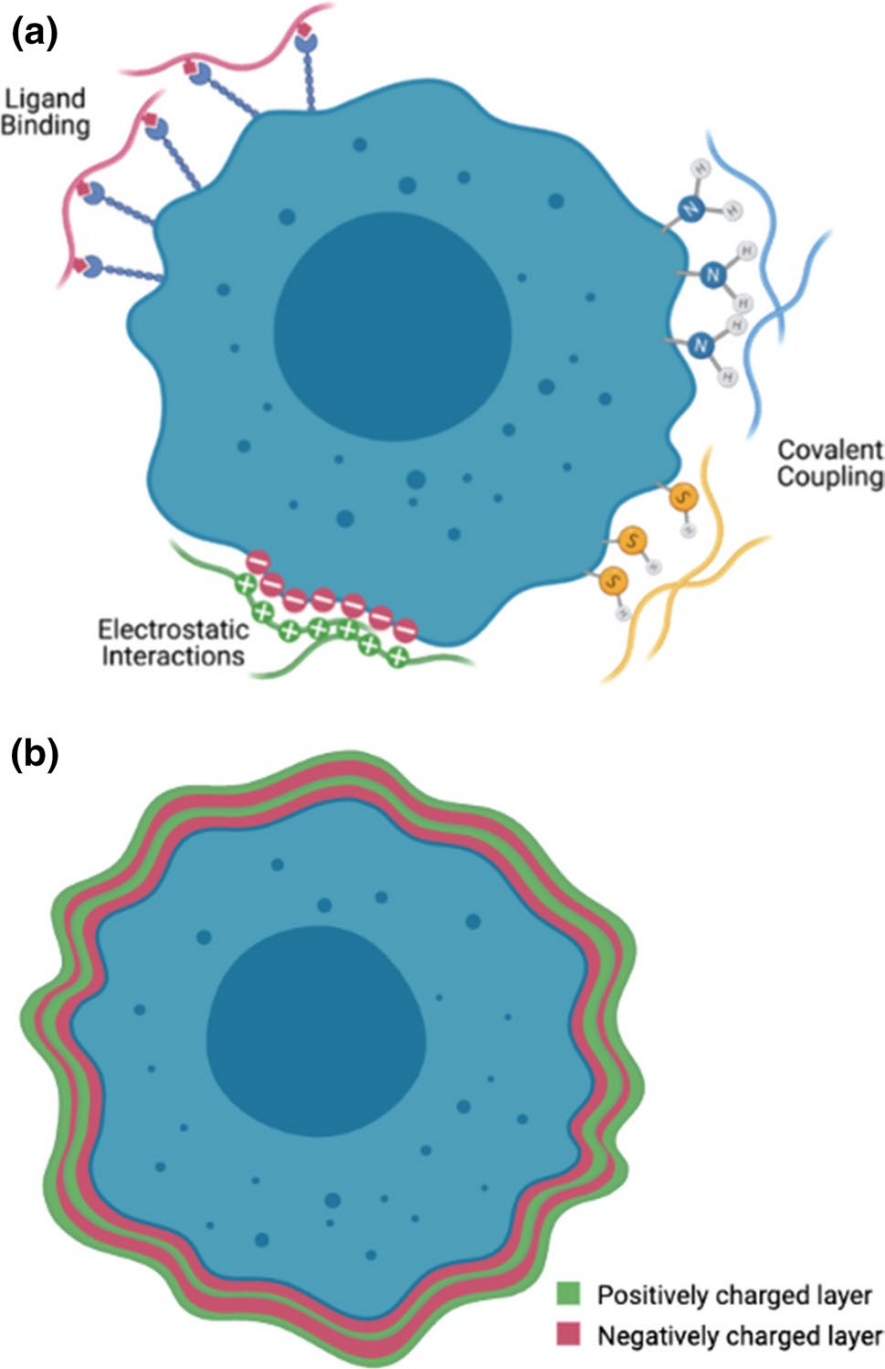
Schematic overview of the different methods to coat cells with polymers. **a** Various interactions to anchor polymers. **b** Layer-by layer coating of a cell with alternating polymers. Consideration needs to be given to maximising the density and uniformity of the coating to ensure complete isolation from the immune system, whilst also maintaining cell viability

To ensure thin films give uniform coverage over the surface of the cell, care needs to be taken to ensure the polymers are anchored to the cell surface (Fig. 3). Gelation or cross-linking is a popular approach to coat cells, and whilst such processes are simple, they often lead to lack of control over the final product. One significant challenge with this lack of control is the high thicknesses of the polymer coating which can reduce the diffusion of the nutrients needed by the cell.

##### Electrostatic attachment

Early work in this field focussed on using non-covalent electrostatic interactions to anchor polymers to the cell. Multiple layers of polymer can be built upon the surface of the particles using alternating charged polymers in a layer-by-layer (LbL) process to produce a thin polymer film [22]. This enables homogeneous coverage of the cell surface as well as precise control over the membrane thickness and diffusion of molecules through the membrane.

The LbL process is commonly used with polyanionic ALG to complex with polycations from natural (chitosan, gelatin) and synthetic sources (poly-l-ornithine, poly-l-lysine, PEG) [18].

This strategy was used recently to encapsulate human embryonic stem cell-derived retinal pigment epithelial cells (hESC-RPE) [23]. The LbL film was assembled based on three layers of poly(alginate) and gelatin. The results showed an improvement in adhesion, survival, and function of the LbL-coated cells over the control hESC-RPE. LbL films can also be crosslinked to tune their structure. In one such study, researchers designed layers based on cationic PEG-gelatin/anionic PEG gelatin both modified with maleimide groups [24]. This multi-layer could be crosslinked with a difunctional thiol crosslinker containing an enzyme responsive linkage. This linkage allowed the release of the cells once in the tumour microenvironment. This research also demonstrated the enhanced viability of cells due to the LbL coating. A recent study investigated the comparison of natural charged polymers in LbL coating compared to synthetic variants and showed significantly improved cell viability in the case of the natural materials, with reductions in viability ranging from 40 to 90% using synthetic coatings [25].

However, the limitations of this electrostatic approach are threefold. First, cationic polymers such as chitosan are cytotoxic and impinge on the long-term viability of the implanted cells [26]. Second, production of an LbL coating is complex and time-consuming, potentially impacting the viability of cells used for encapsulation [14]. Third, electrostatic interactions can occur between the membrane and nutrients/therapeutics. In the case of insulin release, cationic materials will electrostatically interact with the negatively charged insulin, potentially interfering with the release of insulin from the implant (Fig. 1). To overcome the limitations of electrostatic interactions, LbL systems have been developed that employ hydrogen bonding to facilitate the assembly of the multi-layer films [22, 27].

##### Covalent attachment

An alternative approach is to covalently attach the polymer film directly to the cell surface. Polymers can be coupled to proteins on the cell surface using succinimidyl ester chemistry [28], which forms a covalent amide bond with primary amines from lysine residues. Succinimidyl ester chemistry is readily incorporated into carboxylic acid functional polymers; however, the reaction is relatively inefficient due to hydrolysis of the succinimidyl ester in water. Covalent modification of surface proteins can also affect the function of the proteins, which in turn may have an impact on cell viability.

Other covalent interactions that have been used to drive the coupling of thin films to the surface of the cells include thiol-ene click chemistry [29, 30], and azide/alkyne click chemistry [31]. Thiol-ene chemistry employs alkene-modified polymers, which in the presence of light undergo a photochemical reaction with thiols. Whilst thiols present in proteins are typically present as disulphide linkages, mild reducing agents can be used to generate a larger number of free thiols on the surface of the cell. To avoid affecting the biological function of proteins, polymers can also be anchored to the polysaccharide membrane coating. If cells are fed azido functional sugars, these azides are incorporated into the glycoproteins at the cell surface [31], and into the extracellular matrix [32]. This enables copper catalysed azide-alkyne click reactions (CuAAC) or strain-promoted azide-alkyne click chemistry (SPAAC) reactions with polymers. The advantages of these click reactions are they are bio-orthogonal, so they do not interfere with native biological pathways, and they are highly efficient, which can result in polymer layers of controllable densities [33].

##### Ligand binding

An alternative non-covalent approach is to exploit the naturally occurring adhesive proteins natively expressed on the surface of the cell via the process of ligand binding. Integrins are transmembrane receptors that facilitate cell–cell interactions and also mediate cell signalling [34, 35]. The tripeptide RGD is well known to have a strong interaction with integrin, and by functionalising polymers with RGD, they can be driven to bind efficiently to the cell surface. Mimicking the native cell–cell interactions has the combined benefit of passively modifying the surface of the cell whilst also providing the cells with a signalling environment that better simulates their natural environment.

### Regulated immune response

In addition to preventing the host immune system from recognising the transplanted cells, the polymer membrane also needs to avoid recognition by the immune system itself. Typically, polymers such as PEG are used for the outer coating as it mimics the hydrogen bonding of water. However, it is well established that proteins still adsorb to these PEGylated surfaces and form a protein corona [31, 36]. The composition of the corona is the subject of considerable research interest and a number of groups are attempting to control the composition of the corona to both limit adsorption to the surface, but more importantly limit the adsorption of undesirable proteins like opsonin. One option for controlling these interactions is to deliberately functionalise the surface of the encapsulated cells with native proteins such as human serum albumin [37]. This has the potential to limit the non-specific interaction of proteins to the surface and present a ‘self’ surface to the body. Another approach is to functionalise the surface of the implant with CD47 [38]. CD47 acts as a ‘don’t eat me’ signal on the surface of red blood cells to prevent their clearance from the blood. When red blood cells age, they lose this CD47 and are rapidly cleared from circulation. Functionalisation with CD47 has been employed in nanoparticle research to limit the clearance of nanoparticles and increase their circulation half-life [39].

The final consideration for the polymer surfaces is to ensure that they are suitable for long-term use. The generation of antibodies against the surface can, over time, lead to the rejection of the material [40]. It has been established that humans generate antibodies against PEG [41], which over time will lead to the rejection of the implants. Therefore, there needs to be a focus on engineering PEG to limit the production of anti-PEG antibodies [42], and developing other hydrophilic materials that generate less of an immune response.

### Future avenues for microencapsulation strategies

Current challenges in microencapsulation remain the long-term viability of microencapsulated cells, with the degradation of polymer membranes and pericapsular fibrosis posing issues. In addition, the potential risk of immunogenicity to the polymers remains an issue. Some materials that show promise in this field include polyoxazolines [43], and zwitterionic polymers [44, 45], which have been developed to overcome the immunogenicity of PEG encapsulations.

Polyoxazolines (POX) are non-ionic polymers which display similar properties of high biocompatibility and low non-specific interactions to PEG. The synthetic nature of POX also allows for fine-tuning of its properties similarly to PEG whilst avoiding any immune reactions, a quality demonstrated in studies [46], thus appealing to their use as a stealth polymer for microencapsulation [47].

Synthetic zwitterionic polymers, like polyoxazolines, are neutral in charge but are composed of both cationic and anionic groups promoting a high hydrophilicity which lends them the low non-specific interaction and non-immunogenic properties amongst many others [45, 48]. These properties were exploited in a study which synthesised a hydrogel composed of triazole modified zwitterionic polymers (TR-ZW) to encapsulate and transplant islet cells into a Type-1 diabetic mouse model. The results demonstrated reduced pericapsular fibrosis along with increased vascularization around the islet transplants, whilst inducing normoglycemia for up to 200 days compared to alginate controls [49].

Given that PEG is FDA-approved, it still remains an attractive choice for encapsulation of cells. Thus, an alternative approach lies in derivation of PEG polymers, from a linear configuration into shorter polymers with hyperbranched chains termed poly(oligo(ethylene glycol) methyl ether methacrylate) (POEGMA). The hyperbranched architecture of POEGMA mimics that of a bottlebrush, giving rise to the term polymer brushes. In addition, the dense concentration of polymer branches prevents recognition from the immune system, thus circumventing the issue faced by linear PEG, making POEGMA a stealth polymer, and offering a promising avenue for microencapsulation coating [42].

### Current applications of microencapsulation

**Fig. 4:**
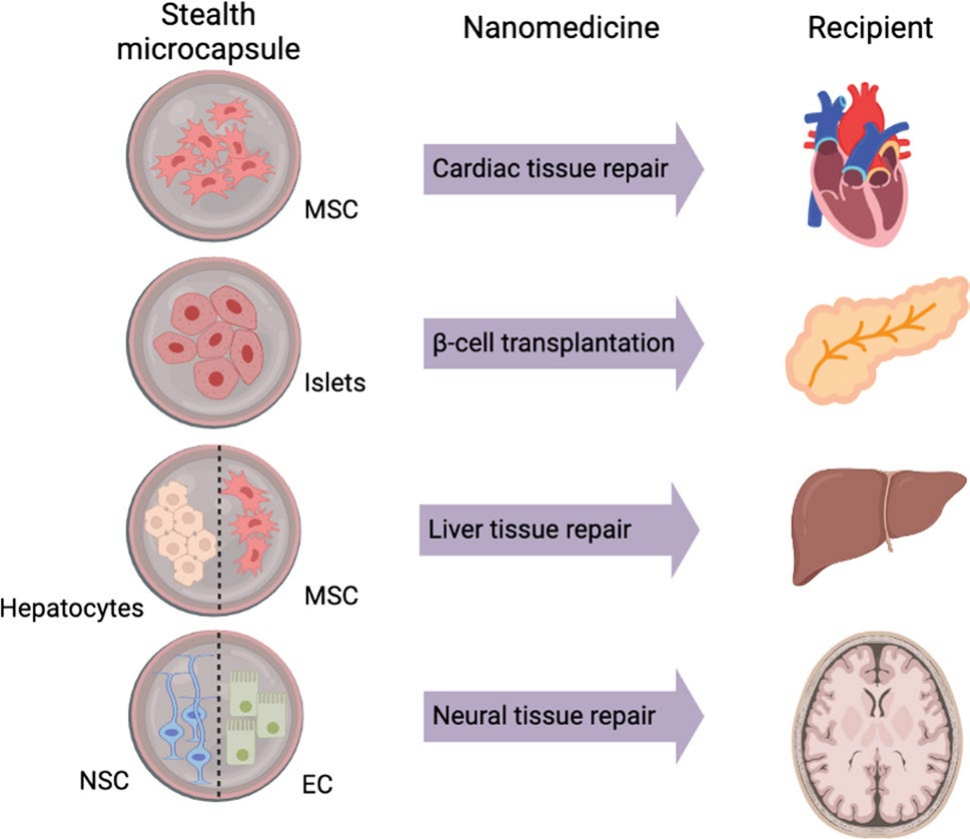
Stealth microencapsulation of cells. Clinical applications of microencapsulation include transplantation of therapeutic cells for repair of cardiac, pancreatic islet, and hepatic and neural tissue. MSC, mesenchymal stem cell; NSC, neural stem cell; EC, endothelial cell

An attractive potential of microencapsulation lies within the ability to perform “stealth” transplantations which may be tailored to fit the disease utilising advances in cell therapies and nanomedicine to produce the cells to be encapsulated (Fig. 4). As such multiple in vivo and clinical trials have been performed to demonstrate the therapeutic capabilities of microencapsulation in various disease models (Tables 1 and 2). The use of alginate, alone or in combination with other polymers, remains a popular choice due to the familiarity of the material; however, alternate natural and synthetic polymers utilised include polyethylene glycol (PEG), agarose, hydroxyethyl methacrylate (HEMA), methyl methacrylate (MMA), or dextrans [15, 50, 51].

**Table 1:** In vivo applications of cell encapsulation

Polymer	Encapsulated cells	Application	References
Natural polymers			
ALG microbeads	MSCs	MI	[58]
ALG-chitosan	(mESCs)-derived cardiomyocytes	MI	[59]
	NPRLCs	ALF	[68]
ALG-PEG	hMSCs	LF	[67]
Gelatin-ALG	hESC-RPE	MD	[15]
Synthetic polymers			
PEGDA	NSCs and ECs	Stroke	[12]
PVA	hMSCs	Bone regeneration	[13]
Star-PEG-vinylsulfone	hiPSC-derived cardiomyocytes	MI	[19]
PEG	Dendritic cells	MS	[74]
TR-ZW	Islet cells	T1D	[40]

ALG, alginate; MSCs, mesenchymal stem cells; MI, myocardial infarcation; ALF, acute liver failure; T1D, type 1 diabetes; ALG-chitosan, Alginate-chitosan; mESCs, mouse Embryonic Stem Cells; NPRLCs, Neonatal Porcine Reaggregated Liver cells; ALG-PEG, Alginate-Poly(ethylene glycol); hMSCs, human Mesenchymal Stem Cells; hESC-RPE, human Embryonic Stem Cell-derived Retinal Pigment Epithelial Cells; MD, macular degeneration; PEGDA, Polyethylene glycol diacrylate; NSCs, Neural stem cells; EC, Endothelial cells; PVA, Poly(vinyl alcohol); hiPSC, human-Induced Pluripotent Stem Cells; PEG, Poly(ethylene glycol); MS, Multiple Sclerosis; TR-ZW, Triazole-zwitterionic polymers

**Table 2:** Clinical applications of cell microencapsulation

Polymer	Encapsulated cells	Application	References
ALG microbeads	Human hepatocytes	ALF	[69]
	CJ-MSCs	PD	[73]
PLO-ALG	Islets	T1D	[48]
APA	Islets	T1D	[49]
PLL-ALG	Islets	T1D	[7]
PLO-ALG	pCPCs	PD	[72]

ALG, alginate; ALF, acute liver failure; CJ-MSCs, Conjunctival Mesenchymal Stem Cells; PD, Parkinson’s Disease; PLO-ALG, Poly-l-ornithine Alginate; T1D, Type 1 Diabetes; APA, Alginate-Polylysine-Alginate; PLL-ALG, Poly-l-lysine Alginate; ALG-chitosan, Alginate-chitosan; pCPs, porcine Choroid Plexus Cells

### Microencapsulation of islets for the treatment of Type 1 Diabetes

**Fig. 5:**
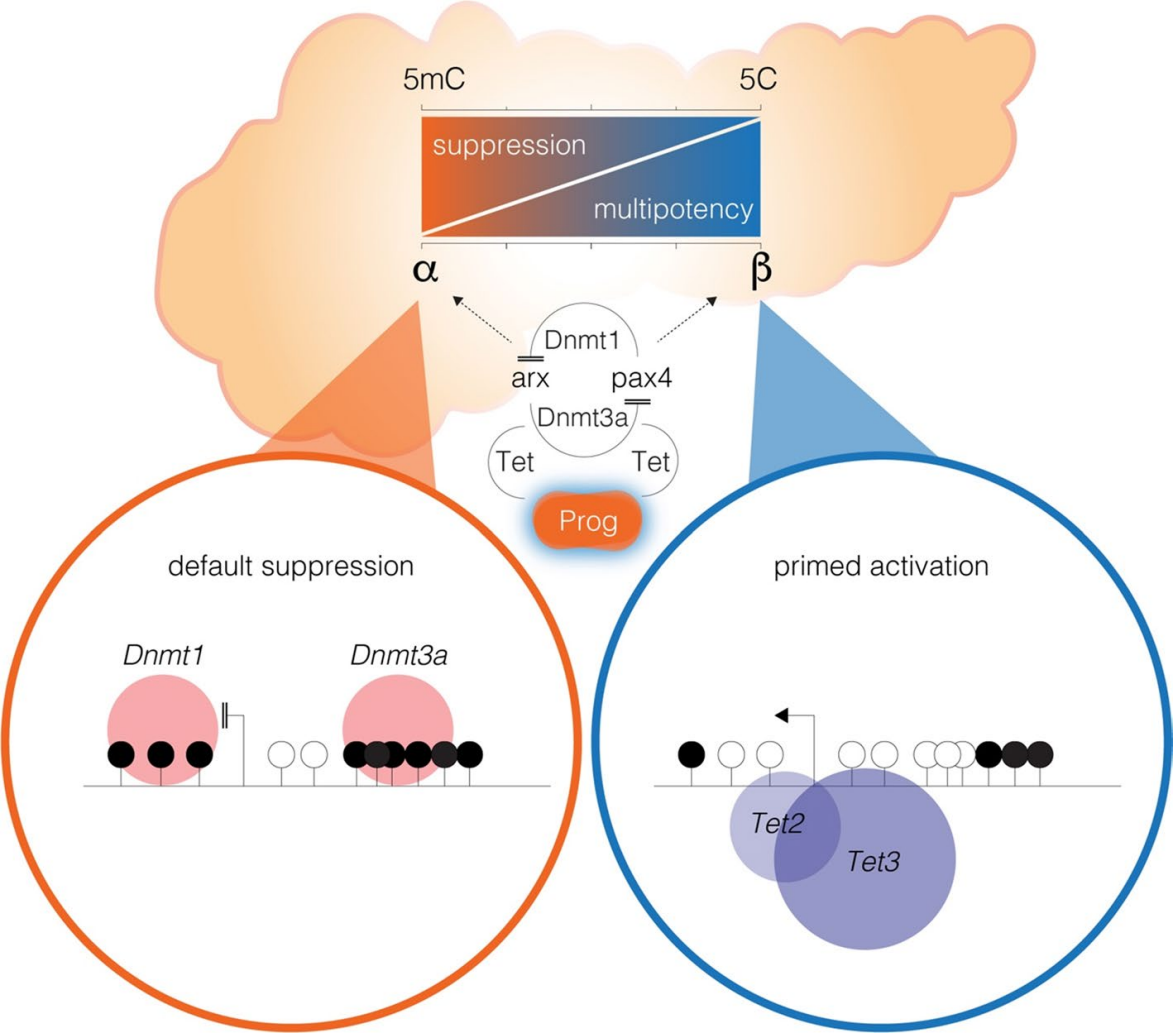
DNA methylation is an epigenetic barrier to reprogramming in the adult pancreas. Islet transition in the pancreas is dependent on DNA demethylation-mediated reprogramming or dmrE. DNA methylation or 5mC by Dnmt writing enzymes are tightly linked with suppression of the reprogramming genes, Ngn3, and Sox11. The loss of DNA methylation (5C) or demethylation is implicated with multipotency of progenitor (Prog) cells and conversion of a-cells and trans-differentiation into b-like cells in the pancreas

Type 1 Diabetes (T1D) is an autoimmune disease that selectively destroys insulin-producing b cells in the pancreas. Even though symptoms usually do not appear before 80% of the b-cell mass has been destroyed, the absolute destruction of these cells leads to the dependence on exogenous insulin administration for survival. Unfortunately, current strategies with insulin infusion are non-physiological, thus supporting the need to develop robust and novel strategies to restore b cells and insulin production to more effectively treat hyperglycaemia. Two solutions aimed at replacing the damaged b-cell mass in diabetic patients exist, such as whole pancreas or islet transplantation. Although efficient, these therapies face the shortage of organ donors together with the associated side-effects of immunosuppressive drugs. A significant challenge for this type of therapy is to find an abundant source of islet cells to transplant into T1D patients to restore glucose homeostasis. There is a major shortage of human islets, and thus, a non-human source of islets (e.g., porcine islets) has also been considered as an alternative approach, although immune rejection remains a major issue. This has been the rationale for developing strategies to protect transplanted beta cells from rejection. With the advances in stem cell and xenotransplantation technologies indicating that an unlimited supply of b-cells or islets could soon be available, there is an urgency to find ways to protect these cells from being killed as a result of transplant rejection. Additionally, endocrine cell reprogramming of progenitor cells into insulin-producing cells provides an alternative new source of glucose-responsive b cells for transplantation [52–54]. We recently showed that influencing epigenetic events is a key condition required to activate developmental genes during b-cell neogenesis, specifically *Ngn3* expressing progenitor cells [54]. Equally important was the finding that the α- to β-like cell conversion induces the re-expression of *Ngn3* in ductal cells and their differentiation into functional insulin cells [54]. We showed that α-to-β-cell conversion by way of directed transcription factor reprogramming, *Ngn3*, and *Sox11* genes undergo dramatic reductions in DNA methylation content which is consistent with re-expression at the mRNA level. Recent in vivo studies propose the *Ngn3* and *Sox11* genes are demethylated during adult β-cell regeneration (Fig. 5). Thus, *Ngn3* appears to be an ideal candidate for strategies that aim to influence DNA demethylation using epigenetic inhibitors, thereby enabling pancreatic β-cell regeneration as a potential path towards improved treatments for T1 and T2 diabetes. Furthermore, 5-aza-cytidine a pharmacological inhibitor of DNA methylation was previously used in the conversion of adult human skin fibroblasts into insulin-secreting cells, indicating that this epigenetic mark represents a barrier to reprogramming [55].

There is growing evidence that encapsulated islets can survive and secrete insulin in vivo and are protected from the host’s immune system. One of the first clinical trials using encapsulated islets was initiated by Calafiore et al. in 2003 on ten Type 1 Diabetes Patients [56]. The outcome of the trial suggested that encapsulated islets can be viable post-transplantation. One of the first examples of xenotransplantation in humans involved the grafting of pig islets that were encapsulated for transplantation [57]. Results from this study underscored the feasibility of using encapsulated islets without the use of immunosuppressants.

The selective permeability of the membrane enveloping the islets of Langerhans is an essential property of microcapsules. It is closely related to the size of the membrane pores. To protect the islets of Langerhans against the host’s immune system, pores must be smaller than immune cells, antibodies, and cytokines. On the other hand, to guarantee the viability and functionality of the islets of Langerhans, the pores must be larger than the size of nutrients, oxygen, therapeutic product, and metabolites [51]. The porosity of the microcapsules can be determined by measuring the permeation rate of known molecular weight molecules (dextrans) towards the inside of the microcapsules or vice versa towards the supernatant [58]. However, these measures are only indicative of the situation in vivo*.* The biocompatibility of microcapsules in the host is an essential element in maintaining the viability and functionality of the graft. For example, the microencapsulated islet graft should minimise or even eliminate the development of a fibrous layer around the microcapsule. Current approaches of encapsulation have not been very successful, since the materials used such as alginate degrade over time and induce pericapsular fibrosis [51].

In recent years, the list of materials used for microencapsulation of the islets of Langerhans has expanded considerably to optimise the bioperformance of the microcapsules [51]. Currently, several types of natural or synthetic polymers are used for microencapsulation of islets, such as agarose, alginate, hydroxyethyl methacrylate (HEMA), methyl methacrylate (MMA), or polyethylene glycol (PEG) [50, 51]. Compared to natural polymers, synthetic polymers have the major advantage of controllable and reproducible chemical and mechanical properties. They can also be synthesised in large quantities more easily than most natural polymers. In vivo studies with PEG microcapsules transplanted in baboons led to insulin-independence for up to 2 years without immunosuppression [59]. A human clinical study was also conducted, which led to a decrease in exogenous insulin intake, albeit for a limited duration [59]. Nanoencapsulation is another approach that involves depositing successive layers of polymers or polyelectrolytes directly on the islets [60]. The goal is to minimise the distance between the islet and the host environment to have a system that is highly responsive to the patient’s insulin needs (Fig. 4). In summary, the rationale for developing cell encapsulation technologies for islet cell transplantation are: (1) to increase the graft survival rate of islets leading to sustainable performance, and (2) to eliminate the need for immunosuppression.

### Microencapsulation in cardiac repair

Cardiovascular disease is a global public health problem leading to myocardial infarction, the major cause of death worldwide. Damage to the myocardium in adults often results in chronic heart failure due to the loss of cardiomyocytes and ineffective tissue regeneration. This has led to efforts at designing cardiomyocyte replacement therapies by cell transplantation or by stimulating endogenous regenerative processes (Fig. 4). Stimulation of endogenous regenerative processes is attractive as it could potentially provide a non-invasive therapy. Cardiomyocytes have been reprogrammed epigenetically using a combination of epigenetic drugs [61]. Remarkably, fibroblasts were able to convert into cardiomyocytes using cardiac-specific transcription factors (Gata4, Mef2c, and Tbx5) and epigenetic remodelling proteins [62, 63]. Lim et al. [64] also found that trichostatin A (TSA, a histone deacetylase inhibitor) can enhance the differentiation of human-induced pluripotent stem cells into a cardiomyocyte lineage suggestive of the functionality of determinants regulated by chromatin modification.

Development of cell transplant strategies is progressing rapidly, and some are being evaluated in clinical trials [65]. The most utilised therapeutics are cardiac progenitor cells, mesenchymal stem cells, cardiac progenitor cells, and extracellular vesicles that are integrated into hydrogels and administered by bulk injection, microencapsulation, and single-cell coating. Unfortunately, the uses of hydrogels themselves have resulted in limited success. Hydrogels break upon cell migration and additionally are degradable. Recently, Levit et al. [66] found that when they microencapsulated human mesenchymal stem cells (hMSC) and transplanted them in a rat myocardial infarction (MI) model, they were able to achieve reduced scar formation and improved revascularisation lending further support for cell-based therapies using microencapsulation platforms. A pre-clinical study using encapsulated pluripotent stem cells soaked in a chitosan micromatrix in an MI model also demonstrated significant enhancement in the cardiac function and survival of animals [67]. Zhao et al., bioengineered an injectable nanomatrix gel containing an amphiphilic peptide and a cell adhesive ligand Arg-Gly-Asp-Ser (PA-RGDS). Upon evaluation of the therapeutic potential and long-term effect of the suspension of mouse embryonic stem cells (mESCs)-derived cardiomyocytes for engraftment in an MI rat model, their results showed retention of engrafted cardiomyocytes for up to 3 months and improved function of the heart post-administration [68].

### Microencapsulation in liver repair

Liver disease can take on multiple forms ranging from the fibrosis associated with cirrhosis to viral hepatitis and acute liver failure. Although the liver has great regenerative capabilities, organ transplantation remains the standard treatment for end-stage liver disease and poses a health burden as only 10% of the global transplantation requirements are currently being met [69]. Thus, cell therapies propose a welcome alternative, with multiple efforts to develop hepatic sources that reduce the requirement for organ donors [70, 71]. A recent experiment demonstrated the Tet1 mediated epigenetic remodelling of ductal cells into hepatic organoids which were then capable of differentiating into cholangiocytes and hepatocytes [72]. Similarly to mesenchymal stem cells and induced pluripotent stem cells [73], human embryonic stem cells when cultured in various hepatic transcription factors such as EGF, FGF-4, and HGF were differentiated into hepatocyte-like cells, which were then encapsulated and demonstrated key enzymatic functions whilst maintaining their viability [74], thus proposing an alternative source for transplantation.

Various in vivo applications of hepatic cell therapies are currently being investigated. The most common microencapsulation approaches to hepatic repair for fibrosis include the utilisation of mesenchymal stem cells (MSCs) which have been shown to secrete anti-inflammatory cytokines, and various growth factors that lead to a reduction in the progression of fibrotic disease whilst preventing immune system recognition when transplanted in ALG-PEG hydrogels [75]. Xenogeneic transplantations of alginate-chitosan encapsulated neonatal porcine hepatocytes into a murine model of acute liver failure also demonstrated similar results, with an improvement in survival rates and liver function following the transplant [76]. In addition, a recent clinical trial transplanted human hepatocytes encapsulated in alginate microbeads in children with acute liver failure, prolonging the duration prior to which a liver transplantation was required and acting as a bridging therapy [77].

### Microencapsulation in CNS repair

Cell therapies for the central nervous system involve the transplantation of cells, as well as immunomodulation. Diseases of the CNS may involve the cellular degeneration and damage of neurons due to various causes such as the degeneration of dopaminergic neurons seen in Parkinson’s disease (PD). Treatment options include prevention of further damage via replacement of dopamine to treat symptoms and more experimentally replacement of neuroprotective factors or cells producing sufficient neurotrophins [13], such as the cells lining the choroid plexus to modify the disease process [78]. The effect of these cells was recently investigated in humans, with the xenotransplantation of porcine choroid plexus cells which were encapsulated in alginate into PD patients [79], in particular their ability to secrete GDNF, VEGF, and BDNF, which are all involved in the promotion of growth and regeneration of neurons. Whilst a mainstay in PD cell therapy has been the use of choroid plexus cells, more novel approaches have included using mesenchymal stem cells harvested from the human conjunctiva and encapsulated using microfluidics with variable results in the efficacy warranting further clinical investigation [80].

Comparably, multiple sclerosis (MS) is an autoimmune disease that results in damage to the CNS due to the aberrant activation of the immune system targeting the myelin sheath of neurons. Sequelae of the disease include progressive paralysis with current immunosuppressive therapies utilised to dampen the disease progression. As such, studies aimed to modulate the inflammatory role of dendritic cells to instead inhibit immune activity. These cells were then encapsulated in PEG hydrogels and injected into murine MS models, with results displaying prolonged survival of the mice and reduced onset of paralysis [81].

## Conclusion

The importance of production methods highlights solubility and bioavailability as critical hurdles to overcome to produce effective nanomedicines. The smarter drug delivery technologies discussed here emphasise targeting and release dynamics are now achieved and aggressively patented. Six focus areas of nanomedicines involve composition, production, and targeting together with nanomedicine triggering and release, and finally method of use. Life sciences are pushing the boundaries in nanomedicine such as the application of synthetic polymers that address the uncertainties in safety and continue to push forward innovation and applied translation. It is envisaged that in the near future, these new developments in polymer encapsulation technology will lead to successful therapeutic outcomes in diabetes and cardiovascular diseases.

## Data Availability

There are no data and materials associated with this review.

## Abbreviations

LbL: Layer-by-layer
CuAAC: Copper catalysed azide-alkyne click reactions
SPAAC: Strain-promoted azide-alkyne click chemistry
ALG: Alginate
PLGA: Poly(lactic-co-glycolic acid)
MSC: Mesenchymal stem cell
hESC-RPE: Human embryonic stem cell-derived retinal pigment epithelial cells
PEG: Poly(ethylene glycol)
POX: Polyoxazolines
POEGMA: Poly(oligo(ethylene glycol) methyl ether methacrylate)
NSC: Neural stem cell
EC: Endothelial cell
ECM: Extracellular matrix
PVA: Poly(vinyl alcohol)
T1D: Type 1 diabetes
HEMA: Hydroxyethyl methacrylate
MMA: Methyl methacrylate
TSA: Trichostatin
PD: Parkinson’s Disease
MS: Multiple sclerosis
